# Direct- versus video laryngoscopy during suction assisted laryngoscopy and airway decontamination (SALAD): A randomized controlled simulation study

**DOI:** 10.1038/s41598-026-60056-1

**Published:** 2026-06-26

**Authors:** Davut Deniz Uzun, Tina Waidner, Benjamin P Zimmermann, Jan C. Havel, Simon Weiss, Tobias Gruebl, Aleksandar R Zivkovic, Markus A. Weigand, Felix C.F. Schmitt, Stefan Mohr

**Affiliations:** 1 Medical Faculty Heidelberg, Department of Anesthesiology, Heidelberg University, 69120 Heidelberg, Germany; 2 Department of Anaesthesiology and Intensive Care Medicine, Bundeswehr Central Hospital, 56072 Koblenz, Germany; 3 Department of Anesthesiology and Intensive Care Medicine, Philipps University of Marburg, 35043 Marburg, Germany

**Keywords:** SALAD; Tracheal intubation, Videolaryngoscopy, Aspiration, Regurgitation, Airway management, Diseases, Health care, Medical research

## Abstract

Pulmonary aspiration during airway management is associated with increased morbidity and mortality. First-pass success during tracheal intubation is a key determinant of patient safety and has been shown to improve with videolaryngoscopy (VL). However, comparative evidence regarding hyperangulated VL and direct laryngoscopy (DL) during massive airway contamination remains limited, particularly within a standardized Suction-Assisted Laryngoscopy and Airway Decontamination (SALAD) workflow. This study aimed to compare DL and hyperangulated VL in a standardized SALAD simulation model. In this 1:1 randomized controlled simulation study, physicians performed tracheal intubation using a standardized regurgitation model. Participants were randomized to hyperangulated VL or DL. The primary endpoint was first-pass success (FPS). Secondary endpoints included time to successful tracheal intubation and time to first successful ventilation. Two hundred physicians from various specialties were enrolled and randomized. FPS was significantly higher in the VL group than in the DL group (94.0% vs. 58.0%; OR 11.34, 95% CI 4.54–28.35; *p* < 0.001). Time to successful tracheal intubation was significantly shorter in the VL group (median [IQR]: 34 s [29–40] vs. 41 s [35–48]; *p* < 0.001). Likewise, time to first ventilation was significantly reduced (median [IQR]: 40 s [34–44] vs. 45 s [40–53]; *p* < 0.001). In multivariable logistic regression analysis, VL remained independently associated with FPS (adjusted OR 13.42, 95% CI 5.18–34.78; *p* < 0.001), whereas age, sex, professional experience, specialty, and additional qualifications were not significantly associated with the primary outcome. In this simulation model of massive airway contamination, hyperangulated videolaryngoscopy significantly improved first-pass success and reduced both intubation and ventilation times compared with direct laryngoscopy during SALAD-assisted airway management. Whether these procedural advantages translate into improved patient-centered outcomes requires confirmation in future clinical studies. Trial Registration The registration of the clinical trial was initiated prospectively on July 15, 2024, in the German Clinical Trials Register (DRKS00034683).

**Supplementary Information:**

The online version contains supplementary material available at 10.1038/s41598-026-60056-1.

## Introduction

Advanced airway management represents a fundamental core competency of anesthesiologists, intensivists, and emergency physicians, as adequate oxygenation can be ensured only when the airway is patent and reliably secured. The process of regurgitation, along with the subsequent occurrence of pulmonary aspiration of gastric contents, constitutes a feared complication in advanced airway management and increases the risk of respiratory complications and mortality^1,2^. The incidence of pulmonary aspiration during general anesthesia is subject to variation, with estimates ranging from 1 in 900 to 1 in 10,000 cases^3,4^. Repeated tracheal intubation attempts further increase the risk of aspiration and hypoxemia, making first-pass success (FPS) a key quality metric in airway management^5,6^. Airway contamination by blood, vomitus, or secretions is a frequent challenge during emergency airway management and may substantially impair visualization and intubation performance^5,7–10^. A standardized and structured approach is imperative for effective advanced airway management, particularly in cases of contaminated airways. In this context, the role of effective suctioning for successful airway management is frequently underestimated^11^. It is evident that adequate suction has the capacity to mitigate a multitude of difficulties that are often precipitated by airway contamination. However, it was not until recently that traditional airway management training incorporated instruction on the simultaneous suction and decontamination of the airway, particularly in cases of large-volume contamination^7^.

The Suction Assisted Laryngoscopy and Airway Decontamination (SALAD) technique was developed to overcome the challenges of a heavily contaminated airway^7^. This method was initially used on training mannequins that had been specially modified to regurgitate simulated contamination material from the esophagus during intubation attempts. Meanwhile, the clinical application of the SALAD technique in vivo has also shown good subjective results^12^. In this context, the role of the laryngoscopy method (DL vs. VL) using the SALAD technique has not yet been sufficiently investigated. In particular, the type of VL blade that should be used has not yet been evaluated in large physician groups. The inherent urgency of tracheal intubations, coupled with the unpredictable nature of pulmonary aspiration, renders the conduct of randomized controlled clinical trials unfeasible.

Although the original SALAD technique was described using Macintosh blade geometry, hyperangulated VL may offer additional advantages in contaminated airways by reducing dependence on a direct line of sight and facilitating glottic identification during ongoing airway decontamination. Therefore, the present study specifically evaluated a hyperangulated VL within a standardized SALAD workflow. Recent multidisciplinary difficult airway guidelines increasingly support the use of VL as a primary airway management technique in both anticipated and unanticipated difficult airway scenarios. In addition to technical device characteristics, these guidelines emphasize the importance of structured airway algorithms, team training, and standardized approaches for improving patient safety during airway emergencies^13,14^. In order to obtain data on this clinically highly relevant topic, the aim of this study is therefore to compare direct laryngoscopy with hyperangulated video laryngoscopy using the SALAD technique in a standardized regurgitation model. The primary objective was first-pass success. Secondary objectives included time to successful tracheal intubation and time to first ventilation. We hypothesized that hyperangulated videolaryngoscopy would improve first-pass success compared with direct laryngoscopy during SALAD-assisted airway management.

## Methods

Between 15th July 2024 and 30th September 2025, two hundred physicians were recruited for this randomized controlled simulation study during in-house and external continuing education events held by the Department of Anesthesiology at Heidelberg University Hospital, Germany. Our hospital is a university maximum care hospital with around 2600 beds. The Department of Anesthesiology is responsible for the provision of comprehensive anesthesiologic services and the oversight of intensive care units, offering a complete range of intensive care therapies, including extracorporeal procedures. The university hospital is home to all the specialist medical departments.

Inclusion criteria were licensed physicians actively involved in clinical airway management who had previous experience with both DL and VL. Individuals who declined participation or provided incomplete baseline data were excluded.

Exclusion criteria were refusal to participate by the participants. Participation in the study was voluntary and free of charge. Informed consent was obtained from all participants before the start of the study. Participants were informed that their performance in the study would have no influence on their course results or other personal consequences. Participants were blinded to the study endpoint; they were only told that the aim was to perform the already familiar SALAD technique quickly and safely. Physicians from various specialties were recruited for the study. All participants were familiar with direct laryngoscopy, videolaryngoscopy, and the SALAD technique prior to study participation. However, previous DL, VL and SALAD-specific training intensity and contaminated airway exposure were not prospectively quantified.

Participants were instructed to perform the already familiar SALAD technique; however, previous SALAD-specific training intensity and case exposure were not quantified and therefore could not be compared formally between groups. All methods were performed in accordance with the relevant guidelines and institutional regulations and in compliance with the Declaration of Helsinki of 1975, as revised in 2013. There was no patient and public involvement for this study. The study was approved by the local ethics committee of the medical faculty at the University of Heidelberg (registration number S-184/2024). The registration of the clinical trial was initiated prospectively on July 15, 2024, in the German Clinical Trials Register (DRKS00034683). This study is reported in accordance with the CONSORT 2025 statement (Supplementary Table 1)^15^.

### Randomization and material

The 1:1 randomization was performed using the online randomizer (randomizer.org Geoffrey C. Urbaniak and Scott Plous). Allocation concealment was ensured using sequentially numbered sealed opaque envelopes prepared by an independent investigator who was not involved in recruitment, enrollment, data collection, or outcome assessment. The intervention group performed tracheal intubation using hyperangulated VL (D-Blade^®^, C-MAC^®^, Karl Storz, Germany), while the control group used DL (HEINE Optotechnik Standard LED laryngoscope with a Macintosh blade 3, Gilching/Germany).

The primary endpoint was first-pass success (FPS). Secondary endpoints included the time to successful tracheal intubation and to the first sufficient ventilation, defined as visible ventilation of the lungs. A size 7.5 endotracheal tube (AEROtube^®^, HUM Luenen, Germany) including stylet was used for the study. A ventilation bag (Spur II, Ambu Germany) was used for ventilation after tracheal Intubation. A rigid suction catheter commonly used for the SALAD technique (SSCOR DuCanto Catheter™ (Medizintechnik werder, Germany)) was used for suction. A commercially available SALAD simulator (S.A.L.A.D. Simulator Nasco Healthcare Inc., Saugerties, NY, USA) was used for the study. To achieve a more realistic appearance, a thickening agent and a brown dye were employed to enhance the regurgitated fluid. Furthermore, a manual pump was used to deliver a standardized amount of simulated vomit of 750 ml.

The selected volume was intended to create a standardized high-contamination airway scenario rather than replicate an average regurgitation event. To our knowledge, no evidence-based standard volume for simulated vomitus currently exists within the SALAD literature. Therefore, 750 ml of simulated vomitus with increased viscosity was used to generate a reproducible and challenging airway contamination model consistent with the educational objectives of the SALAD technique. The manikin’s airway, as well as the tube and stylet, were lubricated before use.

### Definition of outcomes

First-pass success (FPS) was defined as successful tracheal intubation during the first intubation attempt without removal and reinsertion of the laryngoscope blade.

Time to successful tracheal intubation (TTI) was defined as the interval from insertion of the laryngoscope blade/suction device through the teeth until successful placement and cuff inflation of the endotracheal tube.

Time to first ventilation (TTV) was defined as the time to the first visible ventilation of the models lungs.

### Endpoints

The primary endpoint was the FPS rate. Intubation attempts with misalignment of the esophagus were also considered failures. The secondary endpoints were TTI and TTV. Data on the FPS rate, TTI, and TTV were collected by an external investigator who was not involved in the study. Glottic visualization was deliberately not included as an outcome measure in the present study. Multiple prior studies across a wide range of clinical and simulated airway scenarios have consistently demonstrated that VL provides superior glottic visualization compared with DL^16–21^. This advantage is well established and no longer considered a distinguishing or novel endpoint in comparative airway research. In addition, the study was specifically designed to evaluate performance-oriented airway management outcomes, including first-pass success and procedure times, rather than surrogate visualization metrics. Moreover, visualization scores such as the Cormack–Lehane classification were originally developed and validated for direct laryngoscopy and may therefore have limited applicability during videolaryngoscopy. Given the primary objective of assessing procedural performance during SALAD-assisted airway management, first-pass success and time-based outcomes were considered more informative than isolated visualization scores.

### Statistical analysis

A sample size calculation was performed for a two-group, 1:1 randomized design with FPS as the primary binary endpoint. The sample size calculation was based on published studies reporting first-pass success rates for DL and VL in emergency and high-risk airway management settings. Based on these data, conservative first-pass success rates of 60% for direct laryngoscopy and 80% for videolaryngoscopy were assumed for study planning^22^. Utilizing a two-sided alpha level of 0.05 and a desired statistical power of 90%, the requisite sample size was calculated by employing the standard formula for comparing two independent proportions. This resulted in a target enrollment of approximately 109 participants per group. In order to facilitate feasibility the final sample size was set at 100 participants per group, thus yielding a total of 200 participants included in the study. The final sample size of 100 participants per group was determined for logistical and feasibility reasons within the predefined recruitment period. No interim efficacy analyses were performed and recruitment was not terminated on the basis of study outcomes.

A descriptive analysis of all variables was performed, specifying absolute and relative frequencies for categorical variables and median and interquartile range or mean and standard deviation for at least interval-scaled variables. Selected variables were graphically represented using bar charts and box plots, separated into the two groups “direct laryngoscopy” and “video laryngoscopy”. The endpoint variables first-pass success, time to successful intubation, and time to first ventilation were also initially evaluated descriptively, specifying the appropriate measures of location and dispersion for the respective variables. The difference in FPS between the two groups was then examined using a chi-square test at a significance level of 5%. Odds ratios with corresponding 95% confidence intervals were calculated to quantify the effect size for first-pass success. A two-sided p-value < 0.05 was considered statistically significant. This was followed by testing the continuous secondary endpoints (time to successful intubation and time to first ventilation) for normality. Both in the overall cohort and separately for the two groups, there were indications of a skewed distribution (Shapiro-Wilk test *p* < 0.05, as well as a visually skewed distribution in the graphical representation (histogram and Q-Q plot)), so that further evaluation of significant differences between the two groups was performed using the Mann-Whitney U test at a significance level of 5%.

To facilitate interpretation of effect size, mean differences between groups together with corresponding 95% confidence intervals were additionally calculated for the secondary endpoints. Factors associated with first-pass success were further examined using multivariable logistic regression analysis. The model included laryngoscopy technique, age, sex, professional experience, specialty, and additional qualifications. Regression coefficients, standard errors, p-values, odds ratios, and corresponding 95% confidence intervals are reported. Statistical analysis was performed using RStudio version 2025.09.2 + 418.

## Results

**Fig. 1:**
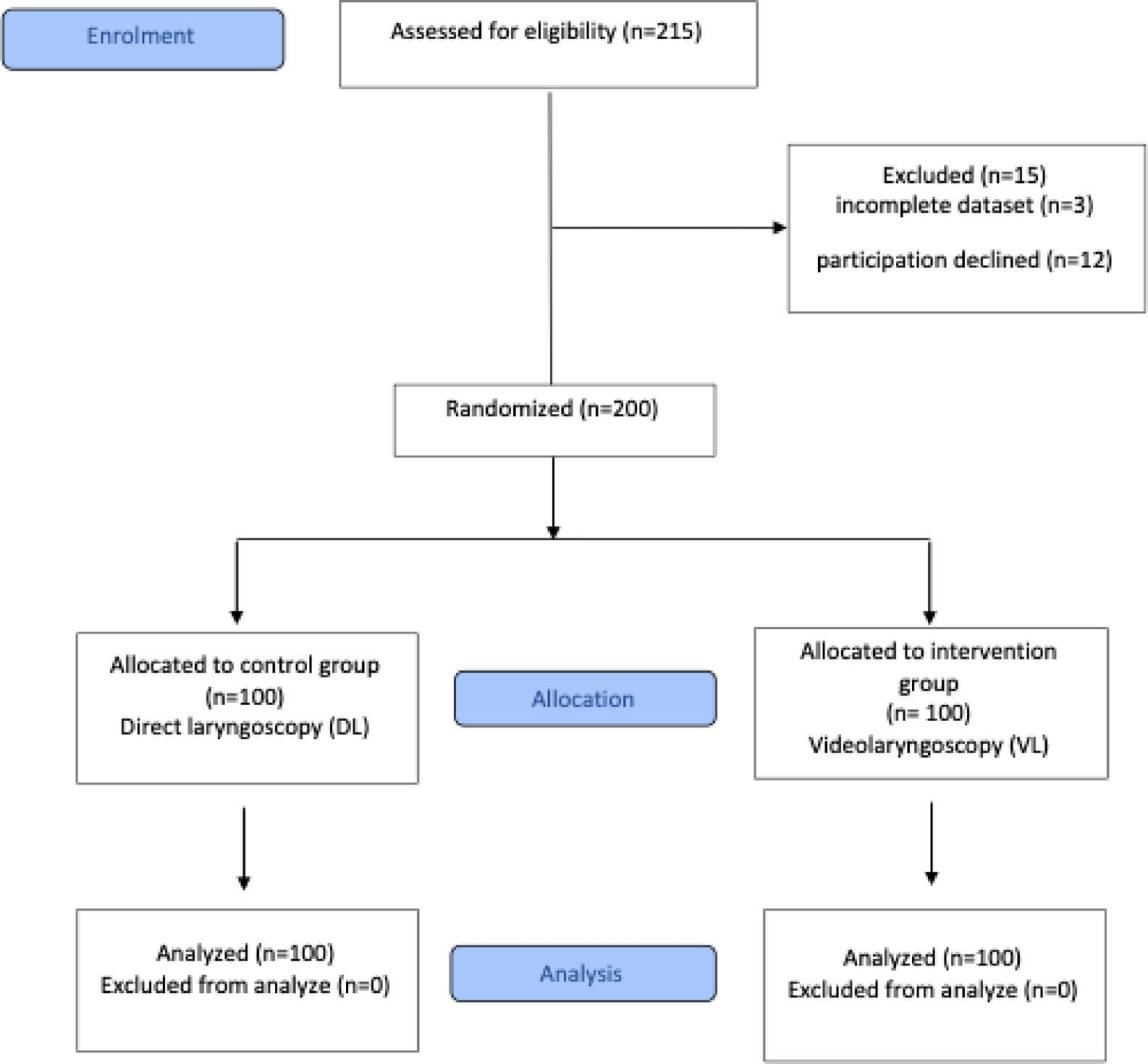
CONSORT flow diagram of participants enrollment and randomization^15^.

A total of 215 individuals were assessed for eligibility; 15 were excluded prior to randomization, including 12 individuals who declined participation and 3 individuals with incomplete baseline data. Two hundred participants were randomized and allocated to either the direct laryngoscopy group (*n* = 100) or the videolaryngoscopy group (*n* = 100). Participant flow through the study is shown in Fig. 1.

**Fig. 2:**
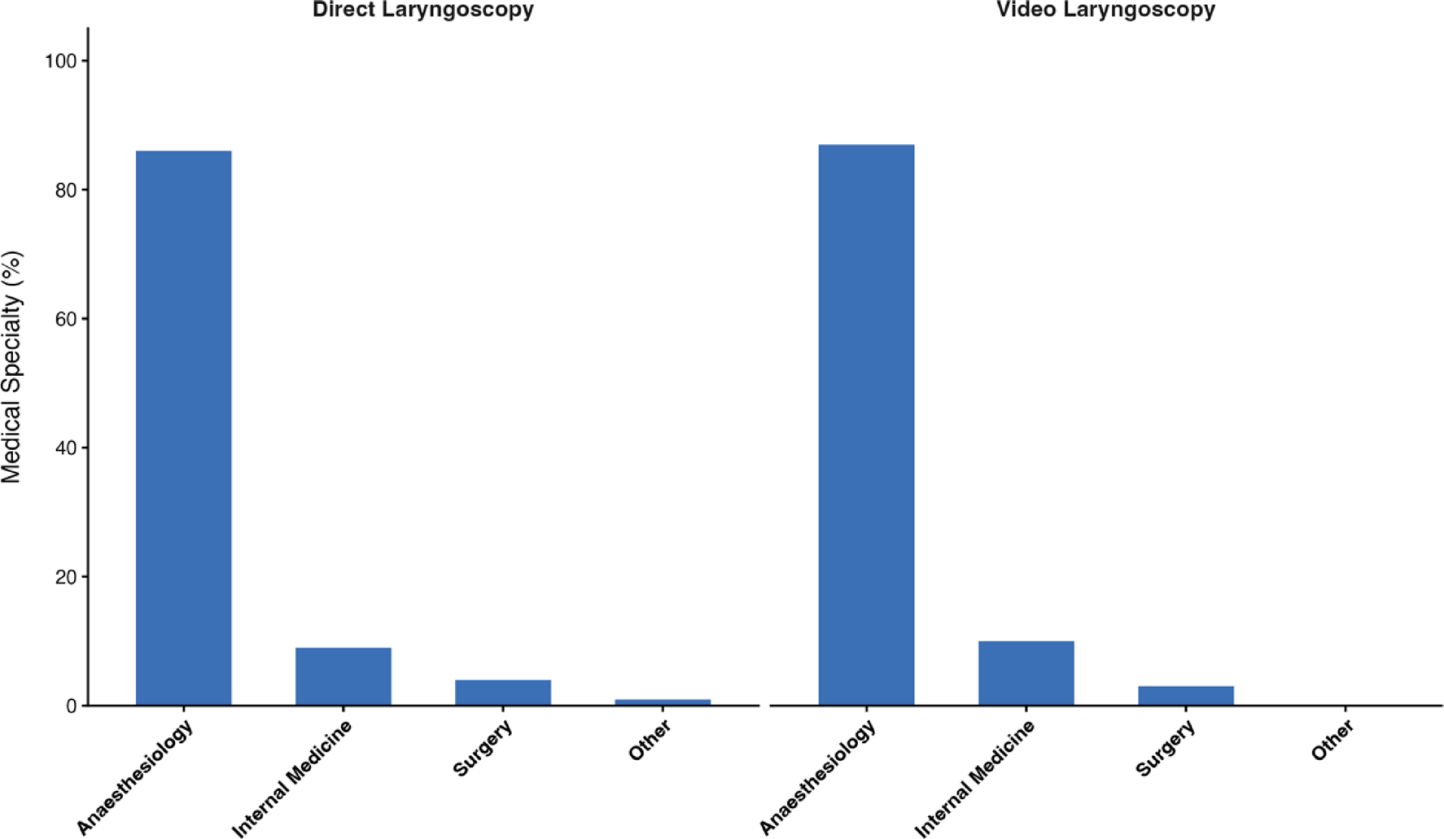
Distribution of participating physicians by medical specialty.

The study population consisted of physicians from various specialties and with differing levels of clinical experience. The majority were anesthetists (DL group: 86 vs. VL group: 87), followed by physicians from internal medicine (DL: 9; VL: 10), surgery (DL: 4; VL: 3), and other specialties (DL: 1; VL: 0) (Fig. 2).

Mean age was 37.42 years in the DL group (minimum 25, maximum 65; range 40) and 36.66 years in the VL group (minimum 26, maximum 59; range 33). Mean professional experience was 9.62 years in the DL group (minimum 0, maximum 39; range 39) and 9.15 years in the VL group (minimum 1, maximum 30; range 29) (Table 1).

**Table 1:** Baseline characteristics of participants.

Characteristic	DL group (*n* = 100)	VL group (*n* = 100)
Age, years, mean ± SD	37.42 ± 8.62	36.66 ± 7.46
Sex, n (%)		
Female	42 (42)	38 (38)
Male	58 (58)	62 (62)
Specialty, n (%)		
Anesthesiology	86 (86)	87 (87)
Internal medicine	9 (9)	10 (10)
Surgery	4 (4)	3 (3)
Other	1 (1)	0 (0)
Additional qualification, n (%)		
None	32 (32)	28 (28)
Emergency medicine	33 (33)	38 (38)
Intensive care	0 (0)	0 (0)
Emergency + intensive care	35 (35)	34 (34)
Professional experience, years, median	8 (IQR 3–13; range 0–39)	7 (IQR 4–13; range 1–30)

First-pass success was significantly higher in the VL group than in the DL group (94.0% vs. 58.0%; OR 11.34, 95% CI 4.54–28.35; *p* < 0.001) (Table 2).

**Table 2:** Primary and secondary outcomes according to laryngoscopy technique.

Outcome	Direct laryngoscopy (*n* = 100)	Videolaryngoscopy (*n* = 100)	Effect estimate (95% CI)	*p* value
Primary outcomes				
First-pass success, n (%)	58 (58)	94 (94)	Risk difference 36% (25–47) OR 11.34 (4.54–28.35)	< 0.001
Secondary outcomes				
Intubation time, s, median (IQR)	41 (35–48)	34 (29–40)	—	< 0.001
Intubation time, s, mean (SD)	44.1 (14.9)	36.2 (13.3)	Mean difference − 7.9 s (− 11.87 to − 3.99)	< 0.001
Ventilation time, s, median (IQR)	45 (40-53.25)	40 (34–44)	—	< 0.001
Ventilation time, s, mean (SD)	50.1 (16.1)	41.6 (13.6)	Mean difference − 8.5 s (-12.63 to -4.33)	< 0.001

**Fig. 3:**
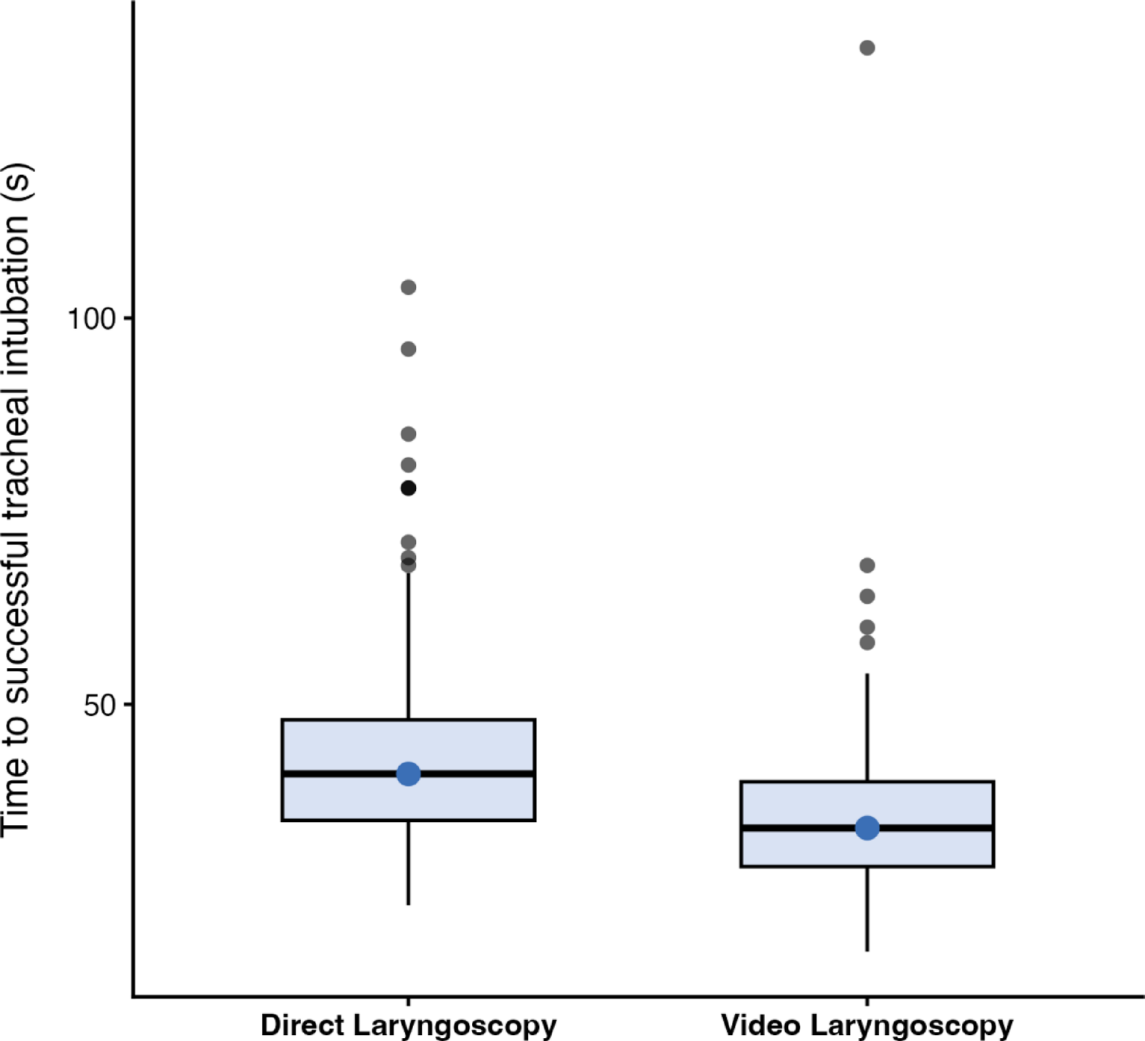
Time to tracheal intubation for direct laryngoscopy and video laryngoscopy.

The time interval from the start of laryngoscopy to successful insertion of the tracheal tube was significantly shorter when VL was used (median [IQR]: DL 41 s [35–48] vs. VL 34 s [29–40], *p* < 0.001) (Fig. 3).

In multivariable logistic regression analysis adjusting for age, sex, professional experience, specialty, and additional qualifications, videolaryngoscopy remained independently associated with first-pass success (adjusted OR 13.42, 95% CI 5.18–34.78; *p* < 0.001). None of the other covariates reached statistical significance (Table 3).

**Table 3:** Multivariable logistic regression analysis of factors associated with first-pass success.

Variable	Adjusted OR (95% CI)	*p* value
Videolaryngoscopy (vs. direct laryngoscopy)	13.42 (5.18 to 34.78)	**< 0.001**
Age, per year	1.13 (0.95 to 1.33)	0.170
Anesthesiology specialty (vs. other)	2.38 (0.82 to 6.91)	0.111
Years of professional experience (per year)	0.88 (0.73 to 1.06)	0.169
Female sex	1.35 (0.61 to 3.00)	0.463
Emergency medicine certification (vs. none)	0.88 (0.34 to 2.28)	0.797
Emergency + intensive care certification (vs. none)	3.06 (0.82 to 11.45)	0.096

## Discussion

In this randomized controlled simulation study involving 200 physicians, the use of hyperangulated videolaryngoscopy within a standardized SALAD workflow resulted in significantly higher first-pass success rates and shorter times to both tracheal intubation and first ventilation compared with direct laryngoscopy. These findings are relevant from an airway management perspective, as repeated intubation attempts in contaminated airways increase the risk of aspiration and hypoxemia, while timely airway control represents a key quality metric in emergency airway management^5,9^. Furthermore the presence of blood or gastric contents in the airway is strongly associated with difficult tracheal intubation and constitutes a significant hazard during emergency airway management^8,23,24^. In the present simulation study, VL was associated with a reduced time to successful tracheal intubation. This finding is not consistently reported in the extant literature. Conversely, numerous studies have documented prolonged intubation times in cases where VL was employed^25^. However, in a multicenter study involving 1,000 patients conducted by Kriege et al., the use of VL and the time to tube placement were also faster in direct comparison to DL (DL 27 s. vs. VL 20 s.)^16^. The observed discrepancies appear to be influenced by a number of factors, including the operator’s experience with videolaryngoscopy, the type of VL, patient-related characteristics, and the specific clinical and environmental context.

Contaminated airways represent a distinct and particularly challenging subset of difficult airway management^26^. Unlike anatomical difficult airways, where restricted glottic visualization is primarily caused by patient-specific factors, contaminated airways are characterized by dynamic obstruction of the visual field through blood, vomitus, or secretions, often in conjunction with time-critical emergency conditions. Consequently, airway management performance depends not only on technical device characteristics but also on the ability to maintain visualization, ensure continuous airway decontamination, and achieve rapid tracheal intubation.

The SALAD technique was developed to systematically manage massive contamination (vomit/blood) through proactive, large-bore suction and simultaneous laryngoscopy. The conceptual advantage is that the suction catheter is not only used intermittently “as needed,” but as an integral part of laryngoscopy, it maintains hypopharyngeal decontamination during tube passage^7^. The existing SALAD literature consists mainly of simulation studies, training interventions, and case reports. Several studies show that SALAD training improves success rates and reduces intubation times, but at the same time, evidence for hard clinical endpoints in real emergency situations remains limited^26^.

In this context, the present study addresses a question that has received minimal attention to date: Which laryngoscopy modality is more effective within a standardized SALAD workflow? Previous studies have frequently examined SALAD as a technology or training package (e.g., pre-/post-training designs) rather than as a direct device or modality comparison study. The observed VL advantage is mechanistically plausible: maintaining a usable line of sight is crucial in contaminated airways.

Beyond technical performance characteristics, airway management success is strongly influenced by human factors. Recent work by Gómez-Ríos et al. highlights the importance of cognitive workload, situational awareness, communication, team coordination, and crisis resource management in airway emergencies^27^. In contaminated airway scenarios, operators must simultaneously perform suctioning, maintain anatomical orientation, manipulate airway devices, and coordinate subsequent ventilation. Consequently, the observed superiority of VL may not only reflect improved visualization but also reduced cognitive burden and improved procedural coordination during complex airway management tasks. This interpretation is consistent with contemporary systems-based approaches to airway management that view successful airway control as the result of both technical and non-technical performance factors.

VL can reduce dependence on a perfect direct line of sight and allows the user to identify more stable glottic landmarks during continuous suctioning. At the same time, coordination between suction catheter, blade, and tube can be complex in SALAD scenarios; VL could offer advantages here through less “head-eye-hand” dissonance and better control of tube passage. This interpretation is consistent with the overarching SALAD evidence, which repeatedly describes “visibility” and continuous decontamination as critical determinants of successful intubation in simulated soiled airway settings^7^.

As expected, the regression showed a strong association between VL use and FPS. Although participants holding both emergency medicine and intensive care certifications demonstrated a numerically higher odds of first-pass success, this association did not reach statistical significance in the multivariable model. These findings suggest that, in addition to the choice of device, the breadth of acute airway and crisis management skills also plays a role. The fact that “years of professional experience” was not significant is consistent with the observation that in rare high-risk situations (massive contamination), specific technical and crisis management skills may be more important than pure time spent in the profession. The difference in first-pass success (36% points) is substantial and typically exceeds the magnitude of many “device comparisons” in standard airway settings^16,17,19^. This finding suggests that contaminated airways are a setting in which the use of VL could be particularly beneficial. Concurrently, external validity should not be overestimated: While simulations do reproduce salient stressors such as visual impairment, time pressure, and coordination requirements, they are limited in their capacity to replicate physiological dynamics, including hypoxia progression, regurgitation variability, anatomical complexity, and secretion viscosity. Additionally, they are unable to fully capture the nuances of team interactions. The extant systematic evidence on SALAD similarly underscores the paucity of clinical outcome data, which is primarily dominated by simulation data^26^. A potential limitation of VL in contaminated airway scenarios is obstruction of the camera lens by blood, vomitus, or secretions. Several studies have reported reduced image quality and impaired visualization under conditions of heavy airway contamination^26^. However, despite this potential disadvantage, VL demonstrated superior first-pass success and shorter procedure times in the present study. Nevertheless, the impact of lens contamination may vary between VL systems and contamination patterns and should therefore be considered when interpreting the present findings.

Although the achieved sample size was slightly lower than originally planned, the observed treatment effect was substantially larger than anticipated during study planning. Although VL demonstrated superior performance in the present study, only a single hyperangulated VL was investigated. The findings therefore cannot be extrapolated to all VL devices. Differences in blade geometry, optical design, tube guidance characteristics, and susceptibility to contamination may result in different performance profiles across VL platforms. Future studies should compare different VL systems, including Macintosh-style devices, in standardized contaminated airway scenarios.

Consequently, the relative robustness of hyperangulated VL versus DL under conditions of extreme or persistent contamination cannot be fully assessed. These considerations underscore the significance of training not only in the SALAD technique itself, but also in predefined escalation strategies, including the rapid transition to alternative airway approaches when visualization deteriorates. Subsequent clinical and simulation studies should systematically evaluate the incidence and impact of VL lens contamination and compare device performance under varying levels of airway contamination to better define the limits of VL in soiled airway management.

## Limitations

Several limitations should be considered when interpreting these findings. First, this was a simulation-based study and therefore cannot fully reproduce the complexity, physiological consequences, and environmental challenges encountered during contaminated airway management in real patients. Consequently, the observed procedural advantages of videolaryngoscopy should not be interpreted as direct evidence of improved patient outcomes. Second, only a single VL model with a hyperangulated blade was evaluated. Therefore, the findings cannot necessarily be generalized to other videolaryngoscopy systems or Macintosh-style videolaryngoscopes.

Third, although all participants had prior experience with both direct and video laryngoscopy, individual differences in previous exposure to videolaryngoscopy and SALAD training may have influenced performance.

Finally, additional esophageal intubation rates, causes of first-pass failure, and the number of subsequent intubation attempts were not prospectively recorded, thereby limiting mechanistic interpretation of the observed differences between groups.

## Conclusions

In this randomized controlled simulation study, the combination of videolaryngoscopy and the SALAD technique resulted in significantly higher first-pass success rates and shorter times to both tracheal intubation and first ventilation compared with direct laryngoscopy. These findings suggest that, within a standardized contaminated airway management approach, videolaryngoscopy may offer procedural advantages over conventional direct laryngoscopy and remained independently associated with first-pass success after adjustment for participant characteristics.

Although these findings were derived from a simulation model and cannot be directly extrapolated to patient outcomes, they support further evaluation of videolaryngoscopy within structured SALAD training programs and contaminated airway management strategies. Prospective clinical studies are required to determine whether the observed improvements in procedural performance translate into improved patient-centered outcomes in real-world practice.

## Supplementary Information

Below is the link to the electronic supplementary material.

Supplementary Material 1

## Data Availability

The datasets used during the current study are available from the corresponding author on reasonable request.

## Abbreviations

DL: direct laryngoscopy
FPS: First-pass success
SALAD: Suction assisted laryngoscopy and airway decontamination
TTI: Time to tracheal intubation
TTV: Time to first ventilation
VL: Video laryngoscopy
